# *Helicobacter pylori* treatment in the post-antibiotics era—searching for new drug targets

**DOI:** 10.1007/s00253-020-10945-w

**Published:** 2020-10-14

**Authors:** Paula Roszczenko-Jasińska, Marta Ilona Wojtyś, Elżbieta K. Jagusztyn-Krynicka

**Affiliations:** 1 Department of Bacterial Genetics, Institute of Microbiology, Faculty of Biology, Univeristy of Warsaw, Miecznikowa 1, 02-096 Warszawa, Poland; 2 Division of Biophysics, Institute of Experimental Physics, Faculty of Physics, Univeristy of Warsaw, Pasteura 5, 02-093 Warszawa, Poland

**Keywords:** *Helicobacter pylori*, Antibiotic resistance, Biofilm, AMP, Probiotics, Therapy

## Abstract

**Abstract:**

*Helicobacter pylori*, a member of *Epsilonproteobacteria*, is a Gram-negative microaerophilic bacterium that colonizes gastric mucosa of about 50% of the human population. Although most infections caused by *H. pylori* are asymptomatic, the microorganism is strongly associated with serious diseases of the upper gastrointestinal tract such as chronic gastritis, peptic ulcer, duodenal ulcer, and gastric cancer, and it is classified as a group I carcinogen. The prevalence of *H. pylori* infections varies worldwide. The *H. pylori* genotype, host gene polymorphisms, and environmental factors determine the type of induced disease. Currently, the most common therapy to treat *H. pylori* is the first line clarithromycin–based triple therapy or a quadruple therapy replacing clarithromycin with new antibiotics. Despite the enormous recent effort to introduce new therapeutic regimens to combat this pathogen, treatment for *H. pylori* still fails in more than 20% of patients, mainly due to the increased prevalence of antibiotic resistant strains. In this review we present recent progress aimed at designing new anti-*H. pylori* strategies to combat this pathogen. Some novel therapeutic regimens will potentially be used as an extra constituent of antibiotic therapy, and others may replace current antibiotic treatments.

**Key points:**

• *Attempts to improve eradication rate of H. pylori infection.*

• *Searching for new drug targets in anti-Helicobacter therapies.*

## Introduction

*Helicobacter pylori*, a Gram-negative spiral-shaped bacterium, is a member of *Epsilonproteobacteria* that specifically colonizes gastric epithelium of humans causing one of the most common infections worldwide. Infection with *H. pylori* affects about half of the world’s population, yet its prevalence varies throughout the world. Currently, incidence rates of *H. pylori* infection remains high in most developing countries, and it is generally related to socioeconomic status and levels of hygiene. Colonization of the stomach mucosa results in various diseases of the upper part of the gastrointestinal tract; however, the disease affects only a subset of infected individuals. *H. pylori* infection elicits gastric mucosal inflammatory responses and causes peptic ulcers (in about 10% of those infected). *H. pylori* is also associated with a high risk for development of mucosa-associated lymphoid tissue lymphoma and adenocarcinoma of the stomach (about 1% those of infected) (De Falco et al. 2015; Cover 2016). In 1994, The World Health Organization classified *H. pylori* infections as class I carcinogens (IARC 1994).

This bacterium was isolated for the first time from human stomach biopsies in 1983, and the first *H. pylori* genome from the 26695 strain was sequenced in 1997 (Marshall and Warren 1984; Tomb et al. 1997). Two years later, *H. pylori* became the first bacterium for which genomes of two strains were compared (Alm et al. 1999). Since then, the number of publicly available *H. pylori* genomes has increased rapidly to more than 1500. There are now (as of 18.05.2020) 1850 *H. pylori* genomes available on PATRIC (Pathosystem Resource Integration Center) (Wattam et al. 2014). These were isolated from patients with various disease symptoms, as well as from a 5300-year-old European natural mummy, named Iceman (Maixner et al. 2016). The *H. pylori* 26695, J99, G27, P12, and N6 are the most commonly used strains in both basic as well as applied research (Ferrero et al. 1992; Tomb et al. 1997; Alm et al. 1999; Belogolova et al. 2013). Comparative genomics experiments have revealed a high level of genomic diversity that results from intra-genomic diversification (for example, point mutations, recombination, and slipped-strand mispairing), as well as inter-genomic recombination, the ability of *H. pylori* to take up exogenous DNA and incorporate it into its genome (Suerbaum and Josenhans 2007; Morelli et al. 2010). While, the average genome size of *H. pylori* is relatively small (~ 1.6 Mbp) and compact (~ 1500 ORF), the high level of diversity can complicate comparative genomics analyses between many strains, and new methods of analysis have to be developed, such as a feature frequency profiling (FFP), an alignment-free analysis approach (Van Vliet and Kusters 2015).

Several international guidelines (Maastricht V Florence Consensus, the Kyoto Global Consensus and the Toronto Consensus Report) elaborated by experts in various fields of microbiology have recommended different drug regimens for treatment of *H. pylori* infections (Sugano et al. 2015; Fallone et al. 2016; Malfertheiner et al. 2017). However, treatment for *H. pylori* still fails in more than 20% of patients, and one would hope to diminish that failure rate to a more acceptable level. The two main obstacles to effective therapy are (1) *H. pylori* resistance to different antibiotics, which correlates with use of antibiotics in the general population, and (2) a lack of patient compliance. Clarithromycin-resistant strains of *H. pylori* have recently been recognized by WHO as one of the 12 priority pathogens for which novel antibiotics are urgently needed (WHO 2017).

**Fig. 1:**
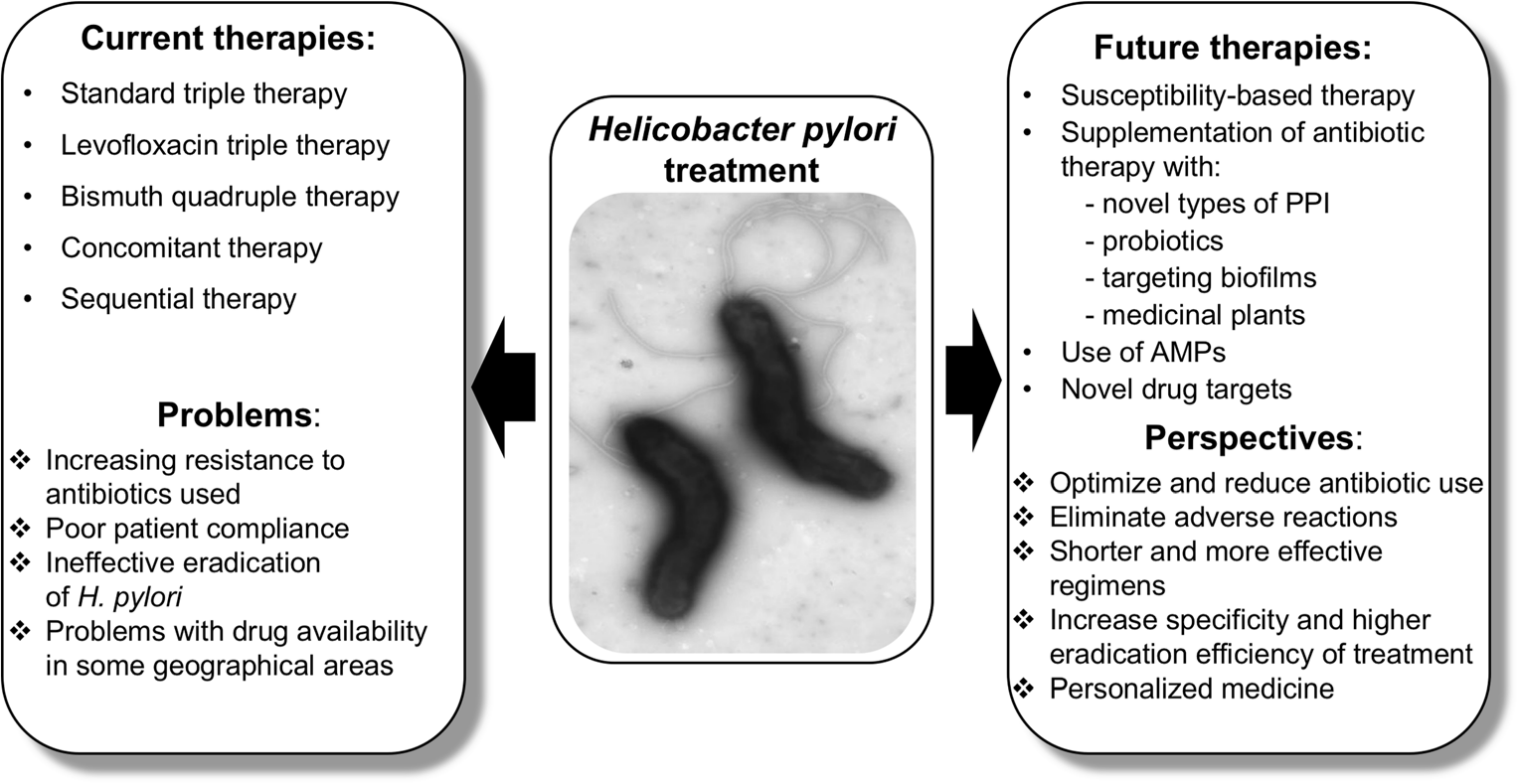
Schematic representation of currently used treatment of *H. pylori* infection and promising future approaches

In this review, we present various novel therapeutic regiments against *H. pylori* that can serve either as a supplement or a replacement for current therapies. Some, such as probiotics, plant extracts, or inhibitors of biofilm formation, should improve eradication rate achieved by standard therapies and should restrict the increase of antibiotic-resistant bacteria. Others have a potential to replace antibiotics. Global screening approaches that combine genomic, proteomic, and metabolic analyses conducted on multiple *H. pylori* strains have led to identification of many possible new drug targets that potentially could eliminate *H. pylori* without interfering with the physiological microbiota. In silico screening that compared *H. pylori* metabolic pathways with those of *Homo sapiens* have indicated proteins unique to the pathogen. Another way to establish new classes of antibacterial agents is targeting virulence rather than the viability of bacteria. In case of *H. pylori*, an understanding of the molecular mechanisms of pathogenesis, in combination with analysis of huge numbers of genomes of strains, responsible for serious disease outcomes, permits selection of a new relevant drug targets. This research concentrates on virulence factors involved in the colonization process and those implicated in gastric cancer development. Solving the structures of many *H. pylori* proteins allows design of effective inhibitors by in silico docking studies. Figure 1 presents principal issues discussed in this review.

## Pathogenesis of *H. pylori* infections

Considerable experimental evidence indicates that the *H. pylori* genotype is a substantial factor determining the type of induced disease (Bridge and Scott Merrell 2013; McLean and El-Omar 2014). However, there are also some host factors, such as polymorphism of the IL-1β, which influences gastric acid secretion, or polymorphism of the CYP2C19, which is the main enzyme metabolizing PPI (proton pump inhibitor), that are associated with the risk of gastric cancer development (Uotani et al. 2015). Some studies indicate that environmental factors, such as nutrition, should additionally be taken into account (Noto et al. 2013; Gaddy et al. 2013).

Analyses of the *H. pylori* pan-genome (defined as the entire gene repertoire in a given species) revealed that this species possesses an open pan-genome. The estimated size of its core genome (the number of genes present in all isolates) varies depending on the number of analyzed strains, their geographical origin, and the methods used; the core genome ranges between more than 1000 to only 244 genes (Salama et al. 2000; Gressmann et al. 2005; van Tonder et al. 2014). Most of the virulence factors are classified as part of the dispensable genome.

*H. pylori* is one of the most common microorganisms able to colonize human gastric mucosa. The bacterium develops many factors or strategies to adapt to this harsh environment and establish persistent infection. Urease, which neutralizes the acidic environment of the stomach, flagella, and a helical shape, in combination with an effective chemotaxis system, aid *H. pylori* to reach the protective mucus layer of gastric mucosa (Sachs et al. 2005; Lertsethtakarn et al. 2011; Sycuro et al. 2012).

Apart from factors involved in the adaptation to acidic stomach conditions, many virulence factors produced by some *H. pylori* clinical isolates engage in the development of disease symptoms. The most extensively studied are those affecting the pathogen’s interaction with host cells, both epithelial and immune system cells, with a consequence of inducing gastric cancer development. This set of virulence factors includes several outer-membrane proteins (OMP) playing a role in bacterial adhesion to gastric epithelial cells (Javaheri et al. 2016; Königer et al. 2016; Moonens et al. 2018), the cytotoxin-associated protein A (CagA) encoded within the *cagA* pathogenicity island (CagPAI) and translocated to eukaryotic cells by type four secretion system (T4SS), and the vacuolating cytotoxin A (VacA). For more details of VacA and CagA biology, we recommend several excellent review papers by Foegeding et al., McClain et al., Tegtmeyer et al., and Backert et al. (Foegeding et al. 2016; Tegtmeyer et al. 2017; Backert and Tegtmeyer 2017; McClain et al. 2017).

Although the majority of gene polymorphisms present in *H. pylori* genomes are distributed randomly, many strains isolated from gastric cancer or peptic ulcer patients possess a specific set of certain gene alleles, e.g., a majority of *cagA* PAI positive strains contain s1 type VacA and type I *hopQ* and produce BabA adhesin (Cover 2016).

## Currently used and recommended antibiotic therapies against *H. pylori*

There are various recommended regimens for *H. pylori* eradication. One of the most commonly used therapies in the world is a standard triple therapy, which consists of a proton pump inhibitor (PPI), amoxicillin (AMX), and clarithromycin (CLR) or metronidazole (MTZ). This regimen should last for 10–14 days to get the best result in *H. pylori* eradication, and it is acceptable only in areas of low CLR resistance (< 15% efficacy). The reason for worldwide increasing resistance to CLR is the high use of macrolides in treatment (Kamboj et al. 2017; Matsumoto et al. 2019). Another regimen due to increasing resistance to CLR is the use of levofloxacin (LVF) triple therapy (LVF, AMX, PPI) because LVF is a broad-spectrum quinolone. However, LVF triple therapy is not recommended as a first-line choice for most patients because resistance to LVF is increasing worldwide, but in areas of low LVF resistance and high CLR and MTZ resistance or unavailability of bismuth, this therapy can be used (O’Morain et al. 2018; Matsumoto et al. 2019; Fallone et al. 2019).

The 2016 Maastricht V/Florence Consensus Report (Malfertheiner et al. 2017) and the 2016 Toronto Consensus (Fallone et al. 2016) recommended the first-line 14-day bismuth quadruple therapy (PPI, bismuth, MTZ, tetracycline (TC)) when there is above 15% CLR resistance or a concomitant therapy (PPI, AMX, MTZ, CLR), if bismuth is not available. The bismuth quadruple therapy is the best choice (especially recommended in cases of penicillin allergy) because it shows effectiveness even against in vitro MTZ resistant strains, and it is well tolerated by patients (Kamboj et al. 2017; Matsumoto et al. 2019; Suzuki et al. 2019; Fallone et al. 2019). There are also new types of bismuth quadruple therapy in which TC or MTZ can be replaced by AMX in areas where TC is difficult to obtain or resistance to MTZ has increased. Sequential therapy is also possible; it consists of PPI and AMX for the first half of therapy (5 days) and PPI, MTZ, and CLR for the second half (Kamboj et al. 2017).

If the first-line therapies fail, different types of second-line rescue treatments for *H. pylori* infection can be used, such as a bismuth quadruple therapy which consist of PPI, bismuth, MTZ, and TC. It is recommended after failure of standard triple therapy (Malfertheiner et al. 2017; Lin and Hsu 2018; Matsumoto et al. 2019). A 10-day TC-LVF quadruple therapy provides the best regimen for a second-line treatment. It consists of an esomeprazole (a type of PPI) and bismuth salts in addition to antibiotics (Lin and Hsu 2018). Furthermore, the 2016 Maastricht V/Florence Consensus Report (Malfertheiner et al. 2017) also recommends fluoroquinolone-AMX triple/quadruple therapy as a second-line treatment. They have comparable effectiveness and consist of LVF, AMX, PPI, and bismuth (only in quadruple therapy).

## Facing resistance of *H. pylori*: novel therapies

Antibiotic resistance of *H. pylori* has reached alarming levels worldwide, prompting an urgent search for more efficient treatments. A recent systematic review and meta-analysis of 178 studies demonstrated an increased rate of primary and secondary *H. pylori* resistance in six World Health Organization Regions (Savoldi et al. 2018; Matsumoto et al. 2019). Success in all antibacterial therapies depends on susceptibility to given drugs, doses, formulations, use of adjuvants (agents that demonstrate additive and/or synergistic effect with antibiotic therapy), treatment duration, and reinfection rates. Antimicrobial susceptibility testing seems to be the best solution to optimize and reduce antibiotics use in *H. pylori* treatment. A meta-analysis conducted by Chen et al., consisting of 13 controlled clinical trials with 3512 participants, showed better efficacy of tailored therapies compared with empirically chosen regimens (Chen et al. 2016). Moreover, establishing *H. pylori* resistance surveillance programs worldwide to provide local resistance reports may be beneficial for preparation of guidelines to treat infection caused by *H. pylori* in particular regions. Matsumoto et al. propose incorporating susceptibility-based therapy against *H. pylori* into practice (Matsumoto et al. 2019). Nonetheless, antibiotic susceptibility testing may also be a challenge; it requires usually biopsy to start bacterial culture. *H. pylori* culture is expensive, intricate, and time-consuming. However, empirical therapies are less effective and face challenges due to the changing profile of *H. pylori* antibiotic resistance across the world. Unnecessary use of antibiotics in *H. pylori* treatment should be avoided because it can influence not only regimen efficacy but also increase the possibility of creating new multi-drug-resistant strains.

### Use of novel components in classical antibiotic therapies of *H. pylori*

One of the new strategies to combat *H. pylori* infections is dual therapy with AMX and PPI. This has demonstrated promising results in eradication of *H. pylori* as well as decreasing the amount of antibiotics used (Suzuki et al. 2019). In the past this approach did not achieve satisfactory outcomes (Bayerdörffer et al. 1995; Koizumi et al. 1998). The successful turning point for this dual therapy seems to be the substitution of conventional PPIs with a novel type of PPI, the potassium-competitive acid blocker, vonoprazan, that has a strong and durable inhibitory effect on acid secretion and generates a neutral environment in the stomach, suitable for *H. pylori* growth. The neutral pH of the colonized niche causes dormant *H. pylori* to enter the replication phase, which makes the bacteria sensitive to antibiotics such as AMX and CLR (Graham and Fischbach 2010). The study of Furuta et al. showed that a 7-day regimen consisting of AMX-vonoprazan provided an eradication rate of 93.8% for *H. pylori* infections (Furuta et al. 2019). The possible limitations of this dual therapy are unavailability of vonoprazan (which is only approved in some countries) and penicillin allergy reactions in some patients (Suzuki et al. 2019).

Rifabutin, a derivative of rifamycin, structurally resembles rifampicin, and it is characterized by a broad spectrum of antibacterial activity. Rifabutin is routinely used as an anti-tuberculosis drug against *Mycobacterium tuberculosis* strains that are resistant to rifampicin, and it is also used against *M. avium* for HIV patients (Gisbert and Calvet 2012). Because this antibiotic exhibits high in vitro activity against *H. pylori* and is not degraded by acid in the stomach, and because rifabutin-resistant *H. pylori* strains have rarely been isolated from patients, rifabutin-based treatment has been considered as a potential option to combat *H. pylori* for more than 20 years*.* The antibiotic inhibits the ß-subunit of RNA polymerase, which is encoded by the *rpoB* gene, and *H. pylori rpoB* mutants that are resistant to rifabutin have been created in the lab (Heep et al. 1999). To date, rifabutin-based triple treatment has mainly been a rescue therapy for difficult-to-treat patients, after multiple failures to eradicate *H. pylori* (Gisbert and Calvet 2012; Ribaldone et al. 2019). A review paper by Gisbert and Calvet used meta-analysis of data published through 2010 to evaluate the role of rifabutin in *H. pylori* treatment. The mean *H. pylori* eradication rate was 73%, but the data in various studies were contradictory and difficult to compare. The observed eradication rates ranged from higher than 80% to 40%, depending on patient status, rifabutin dosage, and duration of treatment (Gisbert and Calvet 2012). A recent study by Graham et al. showed that enrichment of a dual regimen (AMX and PPI) with rifabutin significantly increased treatment efficacy (Graham et al. 2020). Also, the addition of bismuth to a standard rifabutin triple therapy (AMX, PPI, rifabutin) resulted in higher treatment efficacy (Ciccaglione et al. 2016). These results indicate that rifabutin-based therapies have potential as a first- or second-line empirical treatment for *H. pylori*. There are, however, concerns with respect to rifabutin treatment of *H. pylori*: the drug is extremely expensive, severe adverse effects (myelotoxicity, leucopenia) have been observed, and widespread use of rifabutin could cause a surge of resistant *H. pylori* strains, especially because this drug is also commonly used against mycobacteria.

### Antimicrobial peptides in the treatment of *H. pylori*

Antimicrobial peptides (AMPs) have been recently widely verified as an efficient replacement for antibiotics to combat pathogenic microorganism (Sierra et al. 2017; Xu et al. 2020). These compounds are produced by the cells of various organisms as part of their innate immunity, and they provide protection against various pathogens (bacteria, viruses, and fungi), as well as acting as mediators of immune responses (Lei et al. 2019). AMPs usually consist of fewer than 100 amino acids, and they possess an amphipathic structure that allows cationic AMPs to act precisely on anionic bacterial membranes to increase membrane permeability and cause cell death without interacting with the surface of the neutral eukaryotic cell membranes. Apart from acting on cell membranes some AMPs interfere with diverse intracellular processes, such as transcription, translation, or with biogenesis of the cell wall (Mishra et al. 2017).

Neshani et al. searched PubMed, Scopus, and Web of Science databases and found 22 antimicrobial peptides with anti-*H. pylori* activity. The 3 peptides with the strongest anti-*H. pylori* activity were pexiganan, tilapia piscidin 4 (TP4), and PGLa-AM1. These 5-kDa peptides with α-helical structures are cationic and have high positive charge and isoelectric point (Neshani et al. 2019). Most anti-*H. pylori* peptides have the activity against some antibiotic-resistant strains. However, there are some disadvantages to use of AMPs to treat people. Many of natural AMPs can be degraded by both host and bacterial proteases (Pero et al. 2017), and some can cause toxic side effects on human cells. However, the structure of some AMPs has been solved which may allow a synthesis of their analogs lacking cytotoxicity. Additionally, bacteria can become resistant to AMPs after prolonged use (Lei et al. 2019). To avoid AMP degradation engineered probiotic bacteria (lactic acid bacteria-LAB) that colonize the human intestinal tract were tested as a delivery vehicle. However, prolonged release of AMPs by engineered probiotics may kill commensal bacteria and thus may reduce their beneficial effects (Mishra et al. 2017). Despite disadvantages, AMP have synergistic effects in combination with known antibiotics; however, in some cases this may increase their cytotoxic activity. Further in vivo testing is required (Zharkova et al. 2019).

### Probiotics in the treatment of *H. pylori*

Probiotics are defined as “live microorganisms that, when administered in adequate amounts, confer a health benefit on the host” (Hill et al. 2014). They have been used in the prevention and treatment of some gastrointestinal disorders, such as diarrhea, irritable bowel syndrome, and inflammatory bowel disease (Sarowska et al. 2013). Administration of probiotics brings beneficial effects to host microbiota, enhances the immune system, and shows inhibitory effects on pathogens.

Probiotic supplementation is an emerging field in *H. pylori* treatment (Chey et al. 2017). In vitro studies report inhibitory effects of probiotics on the expression level of genes encoding virulence factors, including *ureB*, *vacA*, *flaA*, and *sabA* (Lesbros-Pantoflickova et al. 2007; de Klerk et al. 2016; Urrutia-Baca et al. 2018). Some probiotics, such as *Lactococcus lactis*, *Lactobacillus reuteri*, and *Lactobacillus bulgaricus* produce peptide and nonpeptide antipathogen substances that can inhibit the growth, as well as the adhesion process, of *H. pylori* (Kim et al. 2003; Boyanova et al. 2017; Urrutia-Baca et al. 2018). Moreover, Emara et al. found that using probiotics can improve *H. pylori*-induced histological features by lowering the density of the pathogen on the luminal side of the epithelium, which results in improving the histological inflammatory response that persists even after termination of probiotic supplementation (Emara et al. 2016).

Many clinical studies explored the potential of probiotics in *H. pylori* treatment. They include the effect of a single treatment of probiotics as well as the effect of combining probiotics with standard *H. pylori* therapy (Boonyaritichaikij et al. 2009). However, a recent systematic review by Losurdo et al. based on 11 independent studies suggests that using probiotics alone is not sufficient for *H. pylori* clearance (Losurdo et al. 2018). The use of probiotics in combination with antibiotic therapy appears to be more effective, in both children and adults, compared with a standard therapy alone; however, the increases in eradication rates differ among studies (McFarland et al. 2016; Feng et al. 2017; Wang et al. 2017). Moreover, probiotics can provide a beneficial effect on adverse reactions, such as nausea, vomiting, diarrhea, and taste disturbance, that may occur during eradication therapy for *H. pylori* infections (Tong et al. 2007; Zou et al. 2009; Szajewska et al. 2015; McFarland et al. 2016; Zhang et al. 2017), though the decrease in side effects varied among the studies. The variation may result from different kinds of bacteria and yeasts used as probiotics. Ji et al. suggested that only carefully chosen and examined probiotic strains applied in an appropriate manner have a chance to be effective in *H. pylori* treatment (Ji and Yang 2020). There are still many questions regarding the use of probiotics that need to be explored further, including dosages, duration of probiotic treatment, effectiveness of specific probiotic strains, and the interaction between selected probiotics and antibiotics (Kamiya et al. 2019).

### Phytomedicine in the treatment of *H. pylori*

The use of natural compounds in *H. pylori* treatment is gaining popularity due to their low side effects and low toxicity. Folk medicine, especially traditional Chinese medicine (TCM), constitutes a valuable guide for using plants to combat various diseases. Numerous studies have been performed to evaluate the usefulness of diverse plant extracts in anti-*H. pylori* therapy. Based on in vitro studies (evaluation of MIC—minimal inhibitory concentration), plant extracts were assigned to four categories. The majority were classified as possessing weak-to-moderate activity. An extract of *Impatiens balsamina* L. (*Balsaminaceae*), a Taiwanese folk medicinal plant, showed the strongest activity (Wang et al. 2009).

One patented Chinese drug with two ingredients, *Chenopodium ambrosioides* L. (CAL) and *Adina pilulifera* (AP), is commonly used as a therapy for *H. pylori* infection gastritis and peptic ulcers. Recently, Ye et al. have reported that *Ch. ambrosioides* alone also showed effective antimicrobial activity against *H. pylori*, both in vitro and in vivo. The active ingredients of this plant and mechanism of action have not been elucidated yet, but its inhibitory effect on growth of *H. pylori* is promising for further research (Ye et al. 2015).

Encouraging results from in vitro and in vivo studies were also reported by Kouticheu Mabeku et al., who analyzed the anti-*H. pylori* activity of *Bryophyllum pinnatum*, a plant known for its antiulcer properties. The *B. pinnatum* activity is probably due to active compounds present in the methanol extract, such as phenolics and flavonoids that react with hydroxyl radicals, superoxide anion radicals, and lipid peroxy radicals. They can protect gastric mucosa against reactive oxygen species produced during infection by *H. pylori* (Kouitcheu Mabeku et al. 2017).

Apart from crude plant extracts, purified plant compounds also were tested for their anti-*Helicobacter* activities (Bonifácio et al. 2014). Although the mechanisms of their action require clarification some were recognized as inhibitors of *H. pylori* urease (Xiao et al. 2012; Yu et al. 2015). Structures of the *H. pylori* urease and the urease complex with various ligands have been solved, which allowed a wide range in silico structure-based analysis (molecular docking) of the interactions between this enzyme and plant bioactive components (Xiao et al. 2012; Yu et al. 2015; Hassan and Žemlička 2016). Additionally, several synthetic molecules were also tested. Gains in knowledge may accelerate the design and synthesis of effective inhibitors (for a profound review paper see Kafarski and Talma (2018)).

Combining of phytomedicine with standard medicine may offer effective *H. pylori* treatment. Judaki et al. reported that addition of a turmeric extract (curcumin) to the standard triple therapy significantly increased the eradication rate of *H. pylori* and decreased oxidative damage to DNA (Judaki et al. 2017). Treatment with curcumin reduced the number of bacteria colonizing gastric mucosa and reduced the activity of lipid peroxidases and urease in *H. pylori* infected mice (Kwiecien et al. 2019).

Although many studies of plant extracts or purified plant compounds have been reported, reliable randomized and controlled clinical trials that treat *H. pylori* infections with plant extracts and compare their efficacy with recommended triple therapies are lacking. The only systematic review of clinical trials with TCM conducted between 1982 and 2008 showed an average eradication rate of about 72%. This indicates that plant extract therapy cannot be used as monotherapy, although it has a great potential to assist treatment (Lin and Huang 2009).

## Searching for new drug targets to combat *H. pylori* infection

Discovery of new therapies against *H. pylori* requires identification and validation of novel drug targets. Therapeutic agents are usually designed against basic cellular processes, so the proteins encoded by the core genome are specific and promising candidates as drug targets. Thus, research into *H. pylori* genomics could lead to new therapies. In principle, ideal targets for anti-bacterial agents are *H. pylori* proteins whose homologs are absent in corresponding host and resident microbiota. Various combinations of in silico analyses, together with genomic, proteomic, and metabolomic studies, have been used to identify possible new drug-specific targets. Sarkar et al. used subtractive analysis of metabolic pathways of *H. pylori* HPAG1 and humans and found 25 candidate target proteins that are likely to be essential for viability of the pathogen but have no homologs in human. The largest contributors to the list of potential drug targets in this study were proteins (9 out of 25) involved in the LPS biosynthesis pathway (Sarkar et al. 2012). In a study by Uddin et al. metabolic sets of 61 *H. pylori* strains were extracted and analyzed by pan-genomics and subtractive genomics to reveal 38 non-homologs to human proteins that are potential drug targets. Only one of these was essential for bacterial survival, namely, the saccharopine dehydrogenase that is involved in lysine degradation (Uddin and Khalil 2020). Nammi et al. compared 23 genomes of *H. pylori* with genomes of *Homo sapiens* and 3 others *Helicobacter* species to identify 29 unique proteins that may be potential drug targets; all were essential for *H. pylori* growth and 15 were important for survival of pathogen in the host (Nammi et al. 2016). Dutta et al. used subtractive genomics to identify 10 surface-exposed proteins that may be potential drug targets (Dutta et al. 2006). Screening of 23 genomes of *H. pylori* strains by Nammi et al., in 5-step in silico analyses, revealed 31 genes of interested that are localized within pathogenic islands of *H. pylori* and may be potential drug targets in novel therapies (Nammi et al. 2017). Moreover, pan-genome analysis of global representative of 39 genomes led to characterization of 28 non-host homolog proteins, as universal therapeutic targets (Ali et al. 2015). In summary, some newly identified in silico targets already have been validated and tested by using molecular docking, interactome studies, and structural studies to discover inhibitors that next need to be validated (Sarkar et al. 2012; Nammi et al. 2016; Nammi et al. 2017; Uddin and Khalil 2020). There are also various attempts to find a strategy to develop effective drug delivery systems to eradicate *H. pylori* (El-Zahaby et al. 2014; Verma et al. 2016).

### Targeting biofilms of *H. pylori*

One of the reasons standard therapies are not highly effective in eradication of *H. pylori* may be *H. pylori*’s ability to form biofilms. Since the discovery of this pathogen most studies have focused on the planktonic form (free-living) of *H. pylori*. However, some recent studies indicate that *H. pylori* can develop biofilm structures, both in vitro and in vivo. The presence of biofilms has been observed in gastric mucosa, within glands and gastrointestinal tracts in mice or patients infected with *H. pylori* (Sigal et al. 2015; Keilberg et al. 2016). Most strains of *H. pylori* studied could form biofilms, yet they showed variations in morphology, and some clinical isolates had higher levels of biofilm formation than other strains (Yonezawa et al. 2010). Multiple approaches, including genomic (Wong et al. 2016), transcriptomic (Anderson et al. 2015), proteomic (Shao et al. 2013), and target-specific studies (Cole et al. 2004; Anderson et al. 2015), have been used to identify factors associated with development of biofilms by *H. pylori*; still, the molecular processes need to be elucidated. Bacteria in biofilms exhibit different characteristics when compared with planktonic forms; one of these is an increase resistance to antimicrobial agents (Mah and O’Toole 2001). Recently, an in vitro study by Yonezawa et al. showed that resistance to CLR, MTZ, and AMX increased after *H. pylori* biofilm formation (Yonezawa et al. 2019). Alternative treatment approaches that target *H. pylori* biofilms already have shown some promising results. The use of N-acetylcysteine (NAC), a mucolytic and thiol-containing antioxidant agent, was effective in disrupting biofilm development and prevented formation of new biofilm in vitro and in vivo. Additionally, administration of NAC before traditional antibiotic treatment of patients with a history of at least 4 anti-*H. pylori* therapy failures resulted in a more than 3 fold better eradication rate of *H. pylori* infection (65%) comparing with non-NAC group (20%) (Cammarota et al. 2010).

### Targeting proteins of the primary metabolism of *H. pylori*

Drug targets in *anti-H. pylori* therapy often are proteins involved in the primary metabolism, and their inhibition is bactericidal. There are long lists of targets in this category (see previous subchapter of this article or the paper by Salillas and Sancho). Some of the targets identified in silico have been already tested, and they reveal various level of effectiveness (Salillas and Sancho 2020). The majority of studies described above paid attention to homology of the identified gene products with human proteins but ignored the potential impacts of the proposed therapy on the human microbiota. Recent studies indicate that the interaction of *H. pylori* with the natural microbiota is extremely complex and must be kept in mind while designing new *anti-Helicobacter* drugs (Pero et al. 2019). Here, we present some examples of proteins belonging in the primary metabolism that are promising candidates as novel drug targets.

**Fig. 2:**
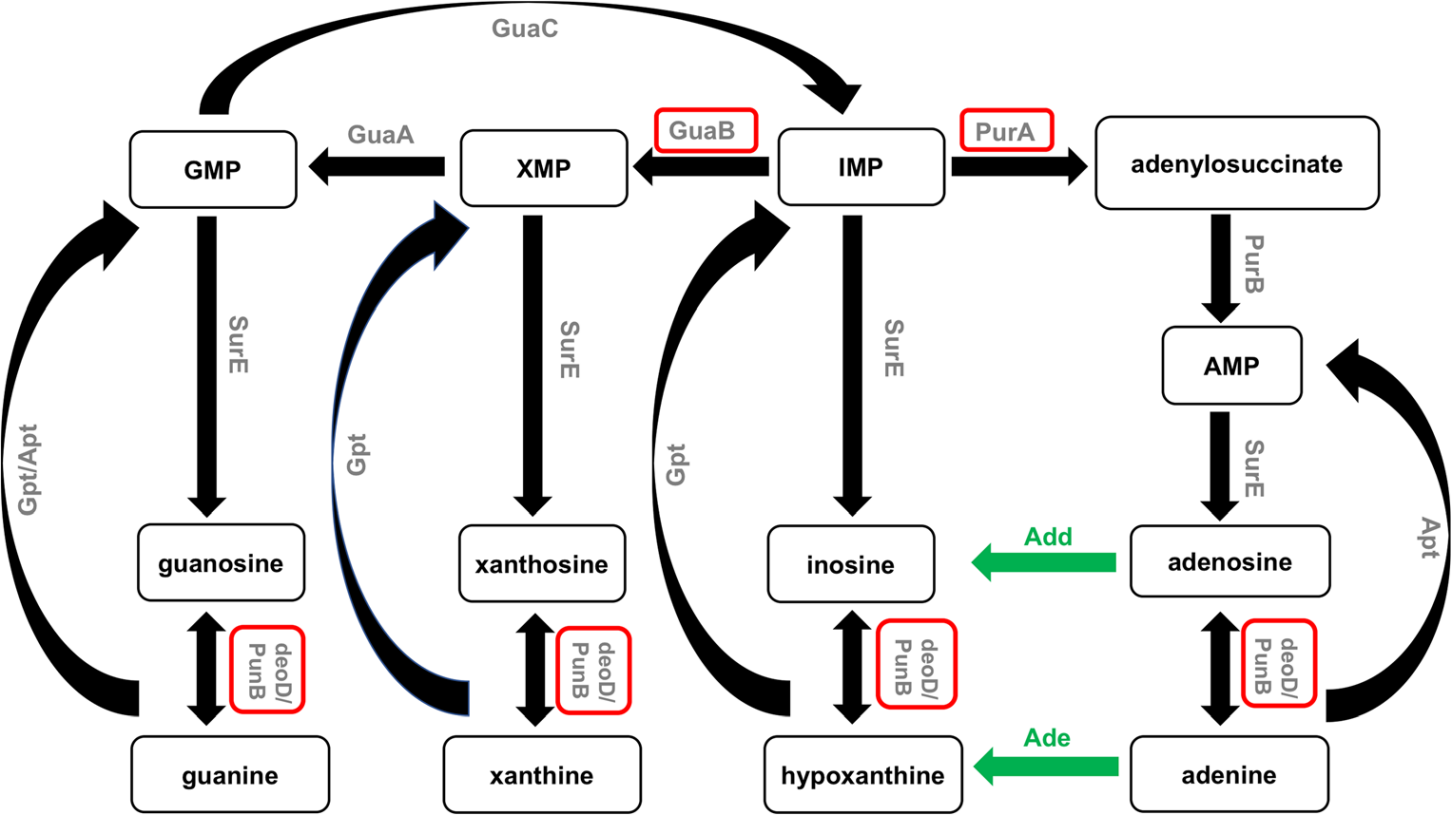
The purine nucleotide biosynthesis pathway in *H. pylori*. Enzymes that have been studied in *H. pylori* by mutant analysis and/or biochemical analysis are shown in gray. Enzymes described in this work are shown in red frames. Enzymes with likely functions, whose genes have not yet been identified, are shown in green (figure adapted from Liechti and Goldberg (2012) and Miller et al. (2012)). Abbreviations: GuaB, IMP dehydrogenase; GuaA, GMP synthetase; GuaC, GMP reductase; PurA, adenylosuccinate synthetase; PurB, adenylosuccinate lyase; Gpt, hypoxanthine-guanine phosphoribosyl-transferase; Apt, adenine phosphoribosyltransferase; SurE, 5′-nucleotidase; *deoD*, gene encoding purine nucleoside phosphorylase; PunB, purine nucleoside phosphorylase; Ade, adenine deaminase; Add, adenosine deaminase; IMP, inosine monophosphate; XMP, xanthosine monophosphate; GMP, guanosine monophosphate; AMP, adenosine monophosphate

Purine metabolism is the growth-limiting step for both prokaryotic and eukaryotic cells. *H. pylori*, similar to other pathogens that have evolved in close association with their hosts, lacks a de novo purine nucleoside synthesis pathway. Therefore, it obtains purine nucleosides only via the purine salvage route (Liechti and Goldberg 2012; Štefanić et al. 2017). Figure 2 presents the purine nucleotide biosynthesis pathway of *H. pylori*. Purine nucleoside phosphorylase (PNP) and adenylosuccinate synthetase (AdSS) are crucial enzymes of the purine salvage pathway. In recent years, the biophysical and biochemical properties of both enzymes have been extensively studied. Purine nucleoside phosphorylase (PNP) catalyzes, in the presence of orthophosphate as a second substrate, the reversible phosphorolytic cleavage of the glycosidic bond of purine nucleosides (Bzowska et al. 2000). Štefanić et al. reported for the first time the unique structure of this enzyme (Štefanić et al. 2017) that influences its interactions with formycin (Narczyk et al. 2018). Formycin A (an analogue of adenosine) is a well-known inhibitor of hexameric PNPs (Bzowska et al. 2000). The formycin A inhibition constant for PNP from *H. pylori* is about threefold higher than reported for *E. coli* PNP (Narczyk et al. 2018). This compound appears to be a good inhibitor of *H. pylori* PNP, and a good lead compound to design more potent inhibitors, but its effect should still be tested in vivo. Adenylosuccinate synthetase (AdSS) is an enzyme that catalyzes the condensation of IMP (inosine-5′-monophosphate) with L-aspartate to adenylosuccinate. Bubić et al. described the biochemical properties of AdSS from *H. pylori*. They showed, inter alia, that AdSS from *H. pylori* is active in vivo as a dimer even at low concentrations and in the absence of ligands (IMP, GTP, GDP). Monomers and dimers of this enzyme seem not to be in a dynamic equilibrium in contrast to *E. coli* AdSS (Bubić et al. 2018). One of the known AdSS inhibitors is hadacidin, which are a natural antibiotic (the fermentation product of *Penicillium frequentans*) and an analogue of L-aspartate (Tibrewal and Elliott 2011). Hadacidin inhibits the enzyme of *H. pylori* strain 26695 in vitro (Bubić et al. 2018). Unfortunately, hadacidin does not significantly affect the growth of *H. pylori* in vivo. The reason could be that the inhibitor does not penetrate into the cells of these Gram-negative bacteria. Solving the structure of this protein alone or with its substrate will help in designing better inhibitors of this enzyme.

Recently, the bacterial IMPDH, GuaB (inosine-5′-monophosphate dehydrogenase) enzyme, a compound of the purine nucleotide biosynthetic pathway, has become popular as a potential target for eradication multi-drug resistant pathogens such as *Cryptosporidium parvum* or *Mycobacterium tuberculosis*. This enzyme is responsible for the oxidation of IMP to xanthosine 5′-monophosphate (XMP). In subsequent enzymatic reactions, GMP is formed and continues to generate nucleotides for the synthesis of DNA and RNA. Juvale et al. performed kinetic studies on *H. pylori* GuaB and compared the activity of a previously tested benzimidazole-based inhibitor with newly synthetized indole-based small molecules. They found that the indole-based molecules have superiority over previously tested inhibitors. They do not affect activity of the human enzyme IMPDH-II and show a non-competitive inhibition against both Hp-IMPDH substrates, IMP and NAD+. The results of these studies are stimulating the development of better bacterial IMPDH inhibitors to overcome infections (Juvale et al. 2019).

Flavodoxin, which is a small soluble electron transfer protein, has also been recognized as a new therapeutic target against *H. pylori* because it is a constituent of the pyruvate-oxidoreductase complex that catalyzes the oxidative decarboxylation of pyruvate. Blocking this metabolic pathway causes bacterial death. Flavodoxins are present in bacterial cells of various species but are absent in animals and humans. Therefore, potential inhibitors will not cause side effects in patients. Solving the *H. pylori* flavodoxin (Hp-Fld) structure, as compared with other flavodoxin, revealed that it possesses a specific cofactor (FMN) binding site, in which a tryptophan residue is replaced by alanine. As a result, a specific cavity on the Hp-Fld surface is created, and this facilitates a search for molecules that bind there and block enzyme action (Cremades et al. 2005). A high-throughput screening method of a library of 10,000 small organic molecules identified 29 compounds that bind to Hp-Fld and stabilize its structure. Detailed experiments to improve the therapeutic and pharmacokinetic properties of selected compounds resulted in designed molecules that inhibit the in vitro electron transfer to flavodoxin physiological partners and also inhibit growth of *H. pylori* strains, including those resistant to commonly used antibiotics (Cremades et al. 2009). Some of the synthetized molecules also significantly reduced *H. pylori* gastric colonization in a mouse model of infection (Cremades et al. 2009; Salillas and Sancho 2020).

Encouraging results arose from a series of experiments which explored the possibility of using MTAN (5′-methylthioadenosine/ S-adenosylhomocysteine nucleosidase) as a target for novel anti-*Helicobacter* drug. The enzyme involved in quorum sensing, menaquinone synthesis, and 5′-methylthioadenosine recycling to S-adenosylmethionine in most bacteria is not essential. However, in members of *Epsilonproteobacteria*—*H. pylori* and *Campylobacter jejuni*, MTAN plays a critical role in their metabolism. These bacteria utilize the unusual futalosine pathway for the biosynthesis of menaquinone, an essential electron transfer agent. It was shown that the inhibition of this pathway is lethal for *H. pylori* (Wang et al. 2012). As this pathway is absent in humans and in human commensal bacteria MTAN is a promising candidate as a target for new drugs. Several the transition state analogues of MTAN have been designed and tested as potential antibiotics in in vitro and in vivo experiments. Solving the crystal structures of MTAN co-crystallized with several inhibitors provided significant information about inhibitor-target interactions, and this is an essential step in exploring the potential of using the transition-state enzyme analogues as antibacterial drugs. Some of the tested inhibitors were able to penetrate the *H. pylori* cell wall as they inhibited the pathogen growth at low concentration (Wang et al. 2012; Wang et al. 2015; Harijan et al. 2019). Aminofutalosine synthase (MqnE) is another enzyme of the futalosine pathway of menaquinone biosynthesis indicated as a potential target for new antibiotics (Joshi et al. 2019).

The essential global gene expression regulator, HsrA, has been shown recently to be a promising target for drug development (González et al. 2019). HsrA belongs to the OmpR-like response regulator that acts as a transcription activator for itself and many others genes, including those involve in redox homeostasis, nitrogen metabolism, energy metabolism, and protein synthesis processes (Olekhnovich et al. 2014). González et al. carried out affinity binding high-throughput screening (HTS) to test in vitro, 1120 FDA-approved small molecules for their ability to a specifically bind to HsrA and inhibit its function. Eventually, four natural flavonoids, apigenin, chrysin, kaempferol, and hesperetin, were demonstrated to exhibit potent bactericidal activity against three different *H. pylori* strains, including those resistant to antibiotics (CLR and MTZ). Subsequent biophysical and molecular docking analyses were used to investigate the mechanism of inhibition of selected potential therapeutics (González et al. 2019).

### Targeting virulence factors of *H. pylori*

Another way to combat infectious disease is to inhibit virulence rather than bacterial viability. This strategy has some advantages over currently used antibiotic therapies. First, it does not induce pressure to select antibiotic-resistant strains and does not disturb the host microbiota. Second, most of the virulence factors are extracytoplasmic molecules that should be easily accessible to potential inhibitors. Additionally, the structures of many bacterial virulence factors have been solved, so the design of new inhibitors and testing their mechanisms of action can be investigate in silico using molecular docking. These types of inhibitors, with respect of their mode of actions, can be divided into two categories: those blocking a single virulence factor and those blocking global bacterial processes involved in pathogen virulence, such as the two-component system, the quorum sensing system, secretion systems, or post-translational protein modifications (Duncan et al. 2012; Krachler and Orth 2013; Brackman and Coenye 2015; Bocian-Ostrzycka et al. 2017). As *H. pylori* has accompanied humankind for more than 100,000 years, its interdependence with the host is extremely complicated (Moodley et al. 2012). There is an evidence that host colonization by *H. pylori* influences the composition of the microbiota not only in the stomach but also in distantly located organs (Kienesberger et al. 2016). Additionally, some inverse association between *H. pylori* infection and some diseases, such as Barrett’s esophagus and its consequences, or allergic asthma, were also reported (Chen and Blaser 2008; Blaser 2011; Rubenstein et al. 2014). In light of this, therapy that targets virulence factors has great potential, as it will not remove *H. pylori* but only disturb its virulence properties. For *H. pylori*, research in this field is still in its infancy, though both types of inhibitors have been recently tested or indicated as potentially effective.

*H. pylori* has a specific helical shape that enhances microorganism motility through the viscous mucus layer of the stomach. Several proteases, acting on the peptide chain of peptidoglycan, play an important role in cell shape determination (Sycuro et al. 2010; Sycuro et al. 2012). One of them, Csd4, was tested as a target for a new drug created through structure-based design, followed by analysis of the Csd4-inhibitor complex. The inhibitor successfully altered on the shape of *H. pylori* in vitro (Liu et al. 2016).

T4SS and its effector molecule CagA are recognized as key virulence factors responsible for gastric cancer development. Blocking this system in patients infected with *cagA* positive strains should be an efficient treatment. Hilleringmann et al. used HTS strategy to identify a small molecule that blocks CagA (a part of the T4SS apparatus, containing an NTP binding motif), and the molecule prevented CagA from translocation into AGS cells. Moreover, the researchers showed that pretreatment of *H. pylori* with this molecule impaired gastric colonization in mice (Hilleringmann et al. 2006). Also, Shaffer et al. identified two compounds that block CagA and peptidoglycan translocation. One interfered with pilus biogenesis, and the second impaired its activity (Shaffer et al. 2016).

Proteins of the Dsb (disulfide bond) system are present in all bacterial cells and are responsible for generation of disulfide bonds between cysteine residues of proteins. As a consequence, they determine protein structure and function. Dsb proteins represent possible targets for antibacterial molecules because virulence factors of several bacteria require this post-translational modification to achieve their proper structures. Inactivation of *dsb* genes attenuates many pathogens (Smith et al. 2016; Bocian-Ostrzycka et al. 2017; Landeta et al. 2019). The *H. pylori* Dsb system is different from Dsb systems operating in cells of Gram-negative bacteria, so its potential inhibitors should not interfere with the physiological human microbiota (Roszczenko et al. 2012; Bocian-Ostrzycka et al. 2015). Several OMPs that play a role in *H. pylori* adhesion with host cells contain cysteine residues, and their proper conformation depends on the action of Dsb proteins (Pang et al. 2014; Javaheri et al. 2016; Moonens et al. 2016). An *H. pylori* strain lacking the major Dsb oxidoreductase, encoded by *hp0231*, is deficient in CagA translocation into host epithelial cells as Hp0231, among other activities, influences the structure of HopQ, an outer membrane protein that mediates pathogen adhesion to epithelial cells by interaction with CECAM receptors (Javaheri et al. 2016; Königer et al. 2016). Lack of Hp0231 abolishes the *H. pylori*-induced IL-8 production and the development of the hummingbird phenotype in AGS cells (Zhong et al. 2016; Grzeszczuk et al. 2018). Moreover, an *H. pylori hp0231*-deficient strain was not able to colonize the gastric mucosa of mice. Additionally, DsbI, encoded by *hp0595*, another component of the *H. pylori* Dsb system, affects the colonization process (Raczko et al. 2005; Godlewska et al. 2006). Therefore, *H. pylori* proteins of the Dsb system have potential to be used as targets for new drugs.

## Conclusions

Many strategies to develop anti-*H. pylori* therapies have been designed and tested at various levels since this bacterium was first recognized as a human pathogen. Nevertheless, we still do not have efficacious and universal drugs to combat this bacterium. There are still important issues concerning diseases induced by *H. pylori* waiting to be resolved. First, which *H. pylori*-infected people should be treated, a decision that will vary according to geographic regions? Anti-*H. pylori* therapy is recommended for cancer and ulcer patients and for patients with some precancer symptoms. However, the treatment of asymptomatic patients is still controversial. The interaction of *H. pylori* with the human microbiota is complex and should be taken into account when new drugs are introduced into the market. As the outcomes of infection are dependent on the *H. pylori* genotype and on host gene polymorphisms, personalized treatment may be needed. The increase in *H. pylori* strains resistant to antibiotics, mainly clarithromycin, is a substantial medical problem. Recent progress in genome sequencing and comparative genomics indicates several housekeeping gene products, that likely are essential for the pathogen viability but have no homologs in humans; these can serve as targets for small-molecule inhibitors. However, this application requires detailed studies of the genomes of the human microbiota. Another approach to fight *H. pylori* infection is to target its virulence factors. Their inhibition attenuates infection and can serve as an individual therapy or in combination with already used antibiotics. This may increase specificity and produce a higher eradication efficacy of treatment. In another approach, probiotics and plant extracts may be administered as a cocktail with antibiotics. It must be stressed that all novel strategies should be tested in animal models and clinical trials, before they are put into practice.

## Data Availability

Not applicable.
